# RRmix: A method for simultaneous batch effect correction and analysis of metabolomics data in the absence of internal standards

**DOI:** 10.1371/journal.pone.0179530

**Published:** 2017-06-29

**Authors:** Stephen Salerno, Mahya Mehrmohamadi, Maria V. Liberti, Muting Wan, Martin T. Wells, James G. Booth, Jason W. Locasale

**Affiliations:** 1 Department of Biostatistics, University of Michigan, Ann Arbor, Michigan, United States of America; 2 Department of Molecular Biology and Genetics, Field of Genomics, Genetics and Development, Cornell University, Ithaca, New York, United States of America; 3 Duke Cancer Institute, Duke Molecular Physiology Institute and Department of Pharmacology and Cancer Biology, Duke University School of Medicine, Durham, North Carolina, United States of America; 4 Department of Molecular Biology and Genetics, Field of Biochemistry, Molecular, and Cell Biology, Cornell University, Ithaca, New York, United States of America; 5 Department of Statistical Science, Cornell University, Ithaca, New York, United States of America; National Research Council of Italy, ITALY

## Abstract

With the surge of interest in metabolism and the appreciation of its diverse roles in numerous biomedical contexts, the number of metabolomics studies using liquid chromatography coupled to mass spectrometry (LC-MS) approaches has increased dramatically in recent years. However, variation that occurs independently of biological signal and noise (i.e. batch effects) in metabolomics data can be substantial. Standard protocols for data normalization that allow for cross-study comparisons are lacking. Here, we investigate a number of algorithms for batch effect correction and differential abundance analysis, and compare their performance. We show that linear mixed effects models, which account for latent (i.e. not directly measurable) factors, produce satisfactory results in the presence of batch effects without the need for internal controls or prior knowledge about the nature and sources of unwanted variation in metabolomics data. We further introduce an algorithm—RRmix—within the family of latent factor models and illustrate its suitability for differential abundance analysis in the presence of strong batch effects. Together this analysis provides a framework for systematically standardizing metabolomics data.

## Introduction

Metabolomics involves the simultaneous analysis of hundreds of small molecule compounds, or metabolites, in biological systems[1, 2]. Metabolite measurements can provide direct biochemical readouts of cellular and organismal behavior and lead to biological insights that are otherwise unobtainable[2, 3]. Quantitation of cellular metabolites can be measured using high-throughput techniques including Mass Spectrometry (MS) approaches[4–6]. In recent years, applications of metabolomics have proven useful in a variety of contexts ranging from basic biochemistry to human health and disease[7–9].

**Fig 1:**
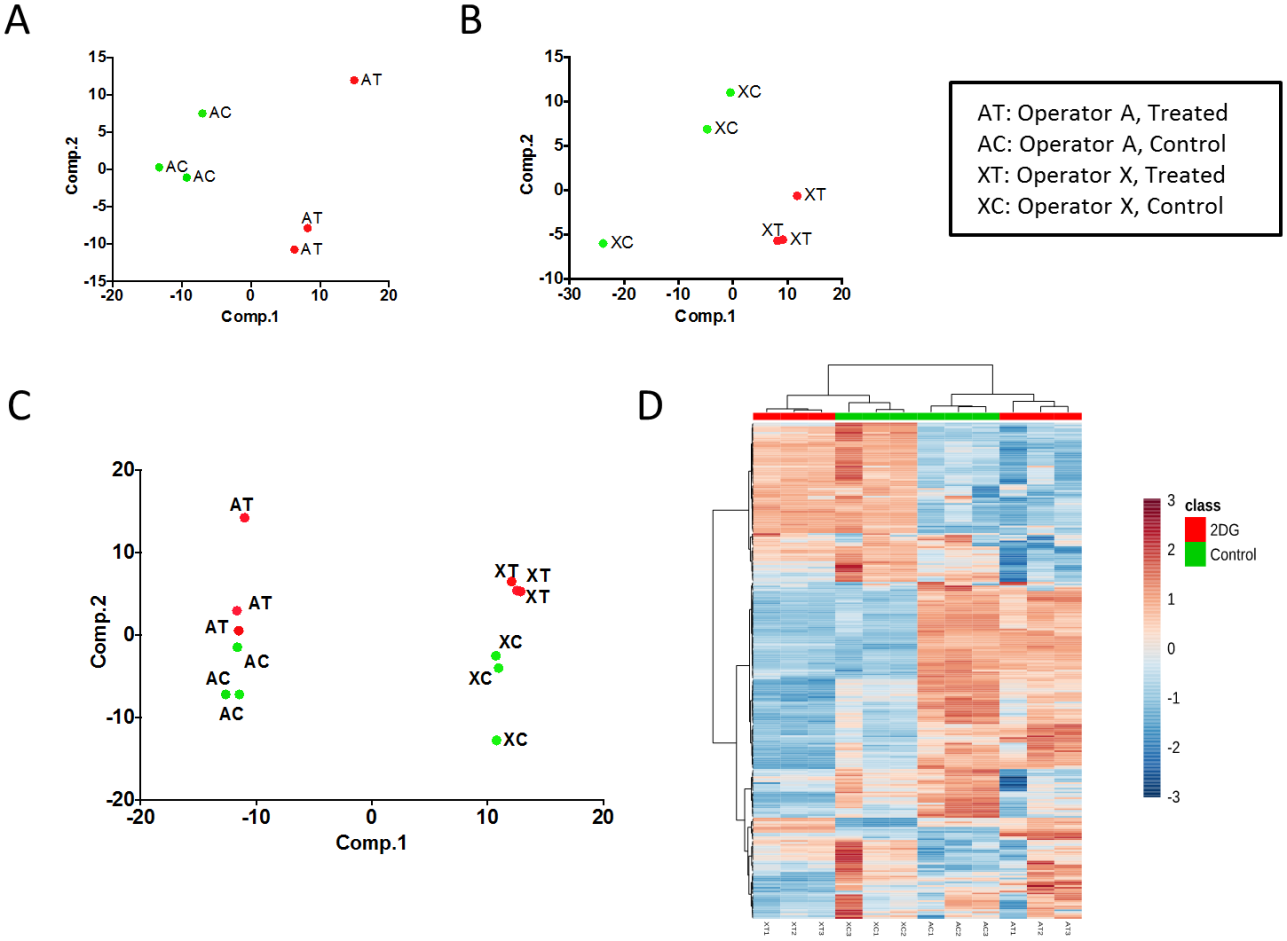
Description of the biological effects and batch effects in the metabolomics dataset used in this study. A) Results of principal components analysis (PCA) on data collected by operator A. Plots illustrate the first two PCs. Samples in green (AC) represent the three replicates from the control condition and samples in red (AT) show replicates from the drug treated condition (2DG = 2-deoxy glucose).B) PCA results as described in (A) using data collected by operator X.C) PCA results as described in (A) on the combined dataset containing samples from both operators A and X.D) Heatmap of metabolite abundances showing hierarchical clustering of the combined dataset. Columns represent samples and rows represent metabolites. Rows are scaled (mean centered and divided by standard deviation).

As with other technologies that acquire high-dimensional data on biological systems, such as gene expression analysis[10], the interpretation of metabolomics data is limited by appropriate mathematical tools for normalization and downstream data processing that ensure reliable and reproducible data collection. Cross-study comparisons and meta-analyses of metabolomics data are currently impractical due to the existence of various sources of unknown experimental, technical, and biological variability. Thus, tremendous advances in metabolomics could be made from the development of standardized algorithms for assessing and removing batch effects from metabolomics data while preserving true biological patterns of interest. We use the term “batch effects” here and throughout, to refer to all undesirable variation in data collected by different operators in different facilities and at different time points. Possible sources of such variability include differences in instrument performance including the current state of the LC column, sample handling, differences in preparation of batches, and many other unmeasurable environmental and technical factors[11] (Fig 1). Removal of this latent variation becomes particularly important when combining data sets to both increase statistical power and improve reproducibility across different metabolomics studies.

Several methodologies are currently available for analyzing metabolite profiles of samples in different conditions to find differentially abundant metabolites, both with and without controlling for potential batch effects. One of the most widely used approaches for assessing differential abundance without the consideration of batch effects is a technique termed Linear Models for MicroArray data (LIMMA)[12]. Originally conceived for microarray gene expression data, LIMMA has grown in applicability to accommodate LC-MS and count-based RNA-Seq data as well[13–15]. However, the consideration of batch effects during analysis with methods such as LIMMA would require addition of parameters into their models, with the ability to do this predicated on directly measuring these effects as covariates. In practice, batch effects present in metabolomics data are often unknown or unmeasurable.

The use of internal standards as controls is one approach that has proven useful for cross-comparison of different batches of samples[16, 17]. However, the variability in the levels of internal control metabolites may not always reflect that of other metabolites due to differences in their chemical properties[18]. Several proposed techniques for the removal of batch effects and the normalization of mass spectrometry-based metabolomics data currently exist in the literature[7, 8, 19, 20]. Many of these methods are based on the use of heavy isotope spike-in quality controls (QCs) or require direct measurement of the possible technical variation through batch factors and/or injection times. The normalization methods work by performing variants of Principal Component Analysis (PCA) on the QC data, and subsequently using the principal components to characterize the unwanted variation. A recent study introduced a normalization algorithm called Remove Unwanted Variation (RUV) for removing batch effects from metabolomics data by taking advantage of reference or control metabolites that are immune to such undesirable variation[11]. Despite its power, this method requires internal standards or prior knowledge about specific metabolites that are robust to treatment effects and can therefore serve as negative controls in the models. Unfortunately, these controls are not always present or straightforward to identify. Other scientists have proposed clustering-based signal drift algorithms using reference samples to correct for batch effects[21, 22]. Brunius et. al cites several alternate strategies for batch effect correction in LC-MS experiments, which include but are not limited to: the utilization of internal standards, normalization with respect to stable feature intensity, more advanced quantile normalization, and quality control sampling. However, while each of these techniques have made significant improvements to the quality of untargeted metabolomics data, certain issues with feasibility are noted as well [citation]. The main drawback to the use of such techniques remains the necessity of prior information which may, or may not, be available to researchers. While internal controls and reference samples are common in LC-MS experiments, there are certain practical limitations in using them in metabolomics experiments. Further, other normalization techniques often do not factor in differences in signal intensity distributions or feature drift patterns, of which are individual experiment-specific. Therefore, there is a need for mathematical methods that do not rely on such internal controls to analyze metabolomics data.

In this study, we apply a range of statistical models to metabolomics data and compare and contrast their performances in differential abundance analyses. We introduce a more heuristic algorithm that provides a hierarchical latent factor mixture model with random primary effect and error variance, referred to herein as RRmix[23] (Random main effect and Random compound-specific error variance with a mixture structure). We show that this class of latent factor models can provide a means to handle unwanted variability without prior knowledge of its source, by instead modeling this systematic experimental noise as a whole, rather than correcting for feature-specific or source-sensitive variation[24]. Instead of attempting to parameterize known effects, factor analysis is used as a robust approach to modeling all hidden factors, without regard to independent metabolite properties or other specific technical artifacts resulting from LC-MS based studies (see Methods). We show that the model-based classification with simultaneous adjustment for unwanted variation[23] provided by RRmix is suitable for handling batch effects in metabolomics data and has advantages over alternative methodologies.

## Materials and methods

### Cell culture and drug treatment

**Fig 2:**
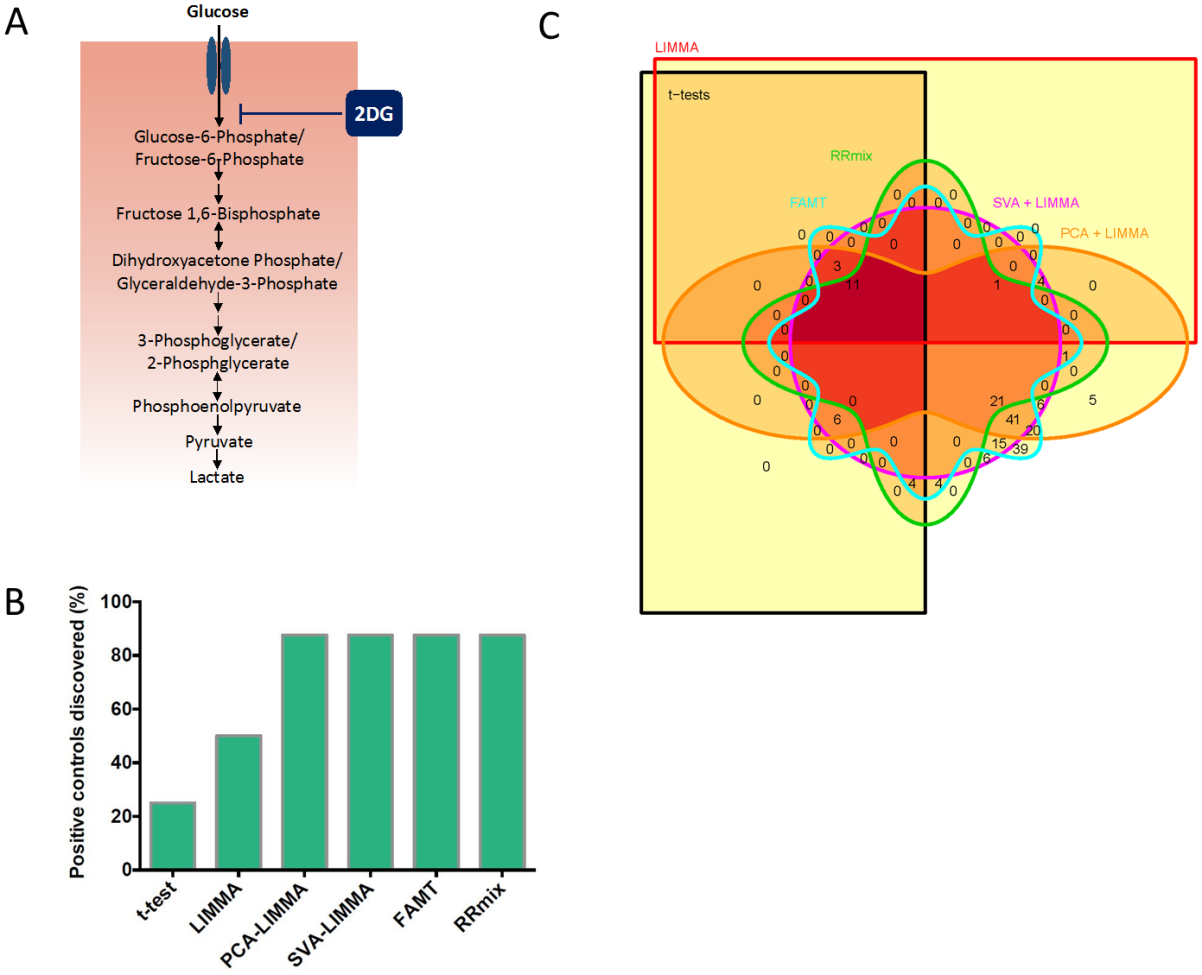
Comparison of performance of 6 methods in differential abundance analysis. A) Schematic depicting the enzymatic steps in glycolysis pathway and the step where 2DG inhibits upon treatment. The metabolites shown in the diagram were analyzed by LC-MS.B) Plot depicting the fraction of positive controls discovered by each method as significantly differentially abundant between the control and 2DG treated samples in the presence of batch effects.C) Venn diagram comparing total number of discoveries made by each of the methods in the combined dataset (at 0.9 posterior probability for RRmix and 10% FDR correction for other methods).

The metabolite samples used for metabolomics were derived from the HCT116 colorectal cancer cell lines. Cells were grown in RPMI 1640, 10% fetal bovine serum, 100 U/mL penicillin, and 100 mg/mL streptomycin. Cells were cultured in a 37°C, 5% CO_2_ atmosphere. At the start of each metabolite extraction experiment, cells were seeded at a density of 3x10^5^ in a 6-well plate and allowed to adhere and grow to 80% confluence. Cells were then washed with phosphate buffered saline (PBS) and treated with either 5mM of 2-deoxy-D-glucose (2DG) (Sigma) or 0.01% DMSO (cellgro) for 6 hours (Fig 2A).

### Metabolite extraction

Metabolite extraction and subsequent Liquid Chromatography High-Resolution Mass Spectrometry (LC-HRMS) for polar metabolite of HCT116 cells were carried out using a Q-Exactive Orbitrap as previously described[5, 25]. For culture from adherent HCT116 cells, media was quickly aspirated and cells were washed with cold PBS on dry ice. Next, 1mL of extraction solvent (80% methanol/water) cooled to -80°C was added immediately to each well and the dishes were transferred to -80°C for 15 min. After, the plates were removed and cells were scraped into the extraction solvent on dry ice. All metabolite extractions were centrifuged at 20,000*g* at 4°C for 10 min. Finally, the solvent in each sample was evaporated using a speed vacuum for metabolite analysis. For polar metabolite analysis, the cell metabolite extract was dissolved in 15μL water, followed by 15μL methanol/acetonitrile (1:1 *v/v*) (LC-MS optima grade, Thermo Scientific). Samples were centrifuged at 20,000*g* for 10 min at 4°C and the supernatants were transferred to Liquid Chromatography (LC) vials. The injection volume for polar metabolite analysis was 5μL.

### Liquid chromatography

An Xbridge amide column (100 x 2.1mm i.d., 3.5μm; Waters) is employed on a Dionex (Ultimate 3000 UHPLC) for compound separation at room temperature. Mobile phase A is 20mM ammonium acetate and 15mM ammonium hydroxide in water with 3% acetonitrile, pH 9.0, and mobile phase B is 100% acetonitrile. The gradient is linear as follows: 0 min, 85% B; 1.5 min, 85% B; 5.5 min, 35% B; 10 min, 35% B; 10.5 min, 35% B; 14.5 min, 35% B; 15 min, 85% B; and 20 min, 85% B. The flow rate was 0.15 mL/min from 0 to 10 min and 15 to 20 min, and 0.3 mL/min from 10.5 to 14.5 min. All solvents are LC-MS grade and purchased from Fisher Scientific.

### Mass spectrometry

The Q Exactive MS (Thermo Scientific) is equipped with a heated electrospray ionization probe (HESI) and the relevant parameters are as listed: evaporation temperature, 120°C; sheath gas, 30; auxiliary gas, 10; sweep gas, 3; spray voltage, 3.6kV for positive mode and 2.5kV for negative mode. Capillary temperature was set at 320°C, and S lens was 55. A full scan range from 60 to 900 (m/z) was used. The resolution was set at 70,000. The maximum injection time was 200 ms. Automated Gain Control (AGC) was targeted at 3,000,000 ions.

### Peak extraction and data analysis

Raw data collected from LC-Q Exactive MS is processed on Sieve 2.0 (Thermo Scientific). Peak alignment and detection are performed according to the protocol described by Thermo Scientific. For a targeted metabolite analysis, the method “peak alignment and frame extraction” is applied. An input file of theoretical m/z and detected retention time of 197 known metabolites is used for targeted metabolite analysis with data collected in positive mode, while a separate input file of 262 metabolites is used for negative mode. M/Z width is set at 10 ppm. The output file including detected m/z and relative intensity in different samples is obtained after data processing. If the lowest integrated mass spectrometer signal (MS intensity) is less than 1000 and the highest signal is less than 10,000, then this metabolite is considered below the detection limit and excluded for further data analysis. If the lowest signal is less than 1000, but the highest signal is more than 10,000, then a value of 1000 is imputed for the lowest signals. After following this data filtering process[5], a total of 265 metabolites remained in the study.

### Statistical analyses of differential abundance

Two independent operators performed the steps described above in triplicates (each operator provided 3 samples from the control and 3 samples from the treated condition), leading to a total sample size of 12. We used six methods for the analysis of this metabolomics data set, both with and without the presence of an operator-specific batch effect. These methods are individual t-tests, LIMMA, Surrogate Variable Analysis followed by LIMMA (SVA-LIMMA), Principal Components Analysis followed by LIMMA (PCA-LIMMA), Factor Analysis model for Multiple Testing (FAMT),[24] and RRmix. By performing simple t-tests on each of the 265 post-processing metabolites, we are provided with a baseline for comparing the efficacy of the other methods.

### Linear Models for MicroArray Data (LIMMA)

LIMMA[12], like the t-test, does not account for latent variation, but it does have several key properties which make it well suited for the analysis of high-dimensional biological data. Firstly, LIMMA calculates a moderated *t*-statistic[12], $\tilde{t}$, which uses a shrinkage estimate of the standard error in the denominator of the t-statistic, utilizing information from all the compounds being analyzed. In addition, the method proposed by Smyth[12] involves closed-form estimates of the hierarchical model parameters, and the proposed moderated *t*, $\tilde{t}$, is more robust to small metabolite-specific sample variance estimation than *t*. Computational implementation of this linear modeling strategy is carried out in the Bioconductor package for R—“LIMMA”[26].

### PCA-LIMMA and SVA-LIMMA

As the issues surrounding the analysis of high-dimensional biological data with batch effects are well known, it is often commonplace for packages such as LIMMA to provide a means for batch effect correction in the pre-processing of the data matrix. The LIMMA-specific function, “removeBatchEffects[13]” provides a variant on the classical mixed ANalysis Of VAriance (ANOVA), to remove any measurable, technical variation not associated with the treatment condition or biological signal of interest. By fitting a linear model with the known batch and treatment effects, the procedure essentially performs an ANOVA decomposition on the data and removes the variability associated with the batches while retaining that which is associated with the experimental design. However, given that the nature of this technical variation makes it often unmeasurable, we explore two methods of batch effect correction, coupled with LIMMA, for the analysis of such affected data: PCA and SVA. In the PCA setting, we first perform a singular value decomposition (SVD) on the row-centered data matrix (M):

$$M_{n\times G}=U_{n\times n}D_{n\times G}V_{G\times G}^{T}$$

where *V* is the *G* × *G* matrix of orthonormal right-singular vectors (eigenvectors) of *M*^*T*^*M*. From there, we extract the first eigenvector from *V*. This is treated as a covariate, i.e. a measured source of variation separate from the biological signal, along with an indicator vector for the treatment condition (differentiation of interest), and is used to build the model matrix used in the LIMMA method.

Surrogate Variable Analysis (SVA)[27] provides an alternative method for accounting for cross-compound dependencies induced by latent variation. The R package, EigenMS [28] implements a variation on this method, with additional functionality specific to mass-spectrometry based metabolomics data, as follows. First, the data is modeled as a function of the predictor variable of interest (i.e. treatment condition), and the matrix of residual values is separated from the effect of treatment status. SVD is then performed on the residuals to obtain eigenvectors. Iteratively, each eigenvector is tested to determine whether or not it is associated with a significant proportion of the residual variation in the data. The subset of metabolites associated with each significant eigenvector is determined through further analysis, and surrogate variables are calculated from this set of eigenvectors and the subset of the original data matrix for the differential metabolites. Finally, these constructed surrogate variables are included in the model along with the primary predictor in the LIMMA model[27].

### RRmix (Random main effect and Random compound-specific error variance with a MIXture structure)

Unlike the closed-form solution found in the LIMMA method, an iterative approach to parameter estimation is defined by Bar and colleagues[29], which adapts a modeling framework for empirical Bayes inference. Computational implementation is carried out utilizing a variant of the Expectation-Maximization (EM) algorithm[30]. The EM algorithm is so named for its two steps at each iteration: the Expectation, or E-Step, and the Maximization, or M-Step. In the E-Step, the expected log-likelihood function for the data is calculated using estimated values for the model parameters. In the M-Step, the Maximum Likelihood Estimate (MLE) of the parameter vector is computed by maximizing the log-likelihood function from the E-Step. The parameter estimates in the E-Step are updated using the MLE from the M-Step, and the algorithm iterates until convergence is achieved. Implementation is both scalable and tractable, and outperforms other computational methods such as Markov Chain Monte Carlo (MCMC) sampling[29].

Wan[23] developed the RRmix model as an extension of a model proposed by Bar and colleagues with an explicit goal of capturing unobserved variation (such as batch effects) through the inclusion of latent factors. In the metabolomics context, the model is defined as follows:

$$y_{g}|\;\beta_{g},\;F_{g},\;\sigma_{g}^{2}=\mu +X\beta_{g}+X_{c}\gamma_{g}+\Lambda \;F_{g}+W_{g}$$

$$W_{g}|\sigma_{g}^{2}∼N_{n}\left(0,\;\sigma_{g}^{2}I_{n}\right)$$

$$F_{g}∼N_{q}\left(0,\;I_{q}\right)$$

$$\beta_{g}|b_{g}∼N_{2}\left(\left[\begin{array}{c} 0\\ b_{g}\psi \end{array}\right],\left(1-b_{g}\right)\left[\begin{array}{cc} \sigma_{0}^{2} & 0\\ 0 & 0 \end{array}\right]+b_{g}\left[\begin{array}{cc} \sigma_{0}^{2} & 0\\ 0 & \sigma_{1}^{2} \end{array}\right]\right)$$

$$b_{g}∼Bernoulli\left(p\right)$$

$$\sigma_{g}^{2}∼IG\left(A,B\right)$$

where *y*_*g*_ is the *g*^*th*^ column of the *n* × *G* matrix of *n* samples of *G* log-transformed, standardized mass-to-charge ratio values, *β*_*g*_ is the *g*^*th*^ column of the matrix *β*_2×*G*_, *μ* is a vector of intercept parameters, *X* = [1_*n*,*x*_] is an *n* × 2 matrix in which the second column is an indicator vector for treatment/control group status, *X*_*c*_ is the *n* × *r* matrix of *r* known covariates, *F*_*g*_ is the *g*^*th*^ column of the *q* × *G* factor matrix, Λ is the *n* × *q* loading matrix, and *W*_*g*_ is the *n* × 1 residual error vector. RRmix is fit simultaneously for all metabolites, and thus, the term *ΛF* is specified such that it accounts for all possible hidden factors contributing to the unwanted variation in the experiment across all compounds. Given that the true factors are unmeasurable and therefore unverifiable, RRmix estimates Λ*F* without the intention of identifying individual sources of latent variation, but rather to capture this variation as a whole. It is the goal of this approach to model Λ*F* in order to enhance the accuracy of differential abundance testing in the presence of a true biological signal. This approach is supported in Leek and Storey[27]. The number of hidden factors (*q*) to be estimated is determined by PCA on the correlation matrix of the data, with subsequent visual examination of a scree plot to determine the number of principle components needed to explain a majority of the variation in the system[31]. Within a reasonable range of values, RRmix is shown in simulation[23] to be robust to the specific choice of *q*.

Given the compound-specific latent indicator (*b*_*g*_), metabolite *g* can be classified as null or non-null using the estimated posterior mean of *b*_*g*,_ or the posterior probability that the compound is differentially abundant between groups. If the estimated *b*_*g*_ $\left({\hat{b}}_{g}\right)$ is greater than a chosen threshold (e.g. 0.9), then it can be concluded that the compound is significantly associated with the group status in *X*. From a modeling perspective, the natural sparsity found in LC-MS data is accounted for by the mixture component, that is, the large number of null effects are accounted for by this approach. The RRmix model is closely related to the Hidden Expression Factor analysis model (HEFT) of Gao et al.[31], although the latter does not include this mixture component. Both RRmix and HEFT are implemented using variants of the EM-algorithm. However, in the case of HEFT, classification is done separately from the fitting process using compound-specific approximate t-statistics.

As RRmix is a Bayesian mixture model, association of metabolite abundance with treatment status is determined using a posterior probability, rather than a classical p-value. As such, RRmix is not directly comparable to the other methods, which rely on post-estimation FDR thresholding. However, it is shown that an FDR less than 0.1 corresponded to empirical posterior probability threshold of 0.9999[23]. We performed a similar calculation in the context of our simulation studies. In the large simulated dataset with two latent factors, a 0.9997 posterior probability threshold resulted in an FDR of 0.1 consistent with the previous finding[23]. In adopting this new threshold with the original data, the total number of significant metabolites discovered by RRmix decreases from 42 (posterior probability = 0.9000) to 22 (posterior probability = 0.9997). However, 6 out of 8 of the positive controls were still recovered (7 out of 8 originally with a 0.9000 cutoff). With regards to specificity, RRmix is robust to the choice of posterior probability threshold due to its simultaneous model selection and estimation and mixture component which accounts for the inherent sparsity of effects found in many high-dimensional biological settings.

### Factor Analysis for Multiple Testing (FAMT)

Another method in the family of latent factor models is Factor Analysis Model for Multiple Testing (FAMT)[24]. The FAMT model is also similar to RRmix but does not include the mixture component or prior assumptions on the regression coefficients and compound-specific variances. As with RRmix, FAMT model fitting is accomplished via the EM algorithm, and as with HEFT classification is done subsequently using compound-specific approximate t-statistics. The sparsity in the data is not modeled directly in FAMT; it is accounted for by a post-hoc FDR thresholding step.

### Simulations

To better understand the comparative performance of the methods detailed above, we have conducted a series of four simulation studies using synthetic data, which closely mirror the LC-MS data set. In the first simulation, two sets of 50 simulated data sets were created, one set with a sample size of *n* = 12 and four latent factors representative of unwanted variability (1) across two machine operators, (2) from analysis on separated but identical LC-MS platforms, and (3–4) from other sources of unknown technical variation, and one set with a sample size of *n* = 6 and no latent structure, representative of the single-operator setting. Within each of the 50 simulated data sets in each set, the data was simulated using model parameters estimated from our original LC-MS data (refer to S1 Text for details). Further, we defined 5% of the simulated metabolites to non-null status between treatment and control groups, and, 5% of the metabolites are simulated so that they resemble negative control metabolites as used previously[11]. Data simulated in the single operator case followed similarly, with no latent factor component and sample size *n* = 6. Due to low sample sizes in the study data, as well as the relatively low metabolite counts, three additional sets of simulations were performed with simulated datasets consisting of 50 and 100 observations on 265 metabolites, 100 and 200 observations on 265 metabolites, and 100 and 200 observations on 500 metabolites, with each pair of observations pertaining to no unwanted variation and two latent factors respectively. As before, the simulation parameters were estimated using the RRmix model analysis on the original data set, and 5% of the metabolites are truly differentially abundant between the samples designated treatment and control. The studies compared the performance of RRmix to several well-known methodologies. Individual t-tests and LIMMA, described previously, represent the analysis of high-dimensional biological data without the consideration of any source of latent variation. RRmix, PCA coupled with LIMMA, unsupervised Surrogate Variable Analysis (SVA), and FAMT, both with and without specifying the number of latent factors, provide means of analysis, which require no additional information or measurements on the sources of technical variation from batches, operators, injection times, or otherwise unspecified.

**Fig 3:**
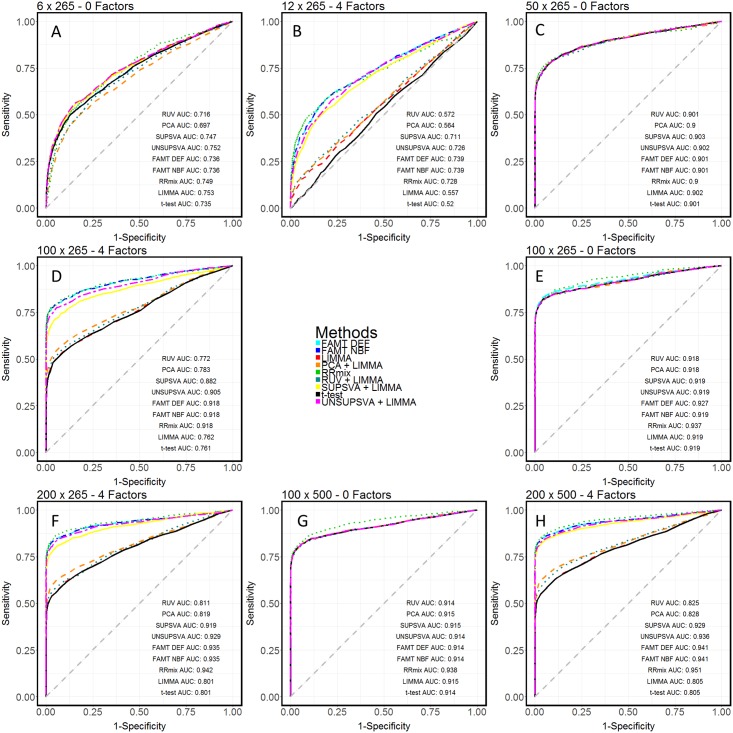
Evaluation of model performance using ROC of simulations. A) Receiver operating characteristic (ROC) plots comparing the performance of the 9 methods in the absence of batch effects with 6 observations and 265 metabolites.B) Receiver operating characteristic (ROC) plots comparing the performance of the 9 methods in the presence of batch effects with 12 observations and 265 metabolites.C) Receiver operating characteristic (ROC) plots comparing the performance of the 9 methods in the absence of batch effects with 50 observations and 265 metabolites.D) Receiver operating characteristic (ROC) plots comparing the performance of the 9 methods in the presence of batch effects with 100 observations and 265 metabolites.E) Receiver operating characteristic (ROC) plots comparing the performance of the 9 methods in the absence of batch effects with 100 observations and 265 metabolites.F) Receiver operating characteristic (ROC) plots comparing the performance of the 9 methods in the presence of batch effects with 200 observations and 265 metabolites.G) Receiver operating characteristic (ROC) plots comparing the performance of the 9 methods in the absence of batch effects with 100 observations and 500 metabolites.H) Receiver operating characteristic (ROC) plots comparing the performance of the 9 methods in the presence of batch effects with 200 observations and 500 metabolites.

**Fig 4:**
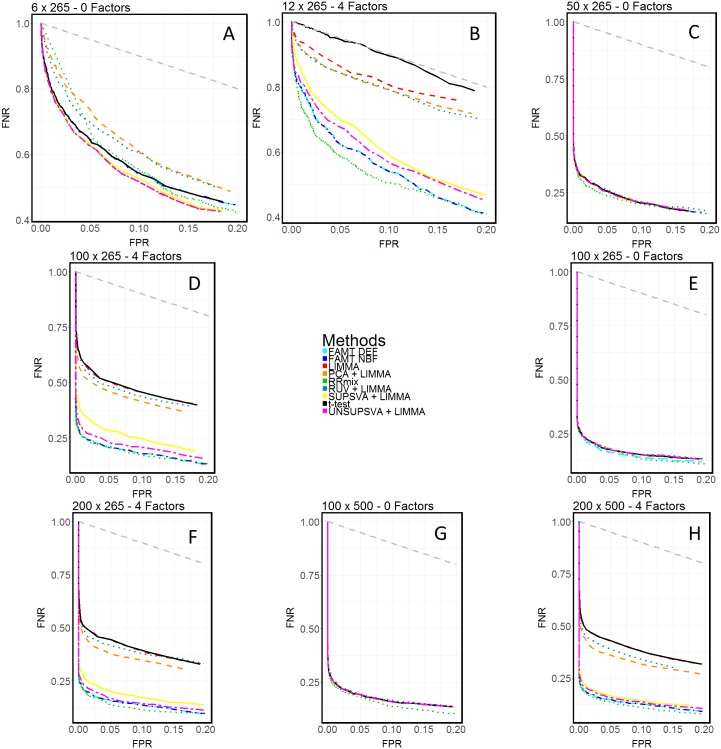
Evaluation of DET using simulations. A) Detection-error tradeoff (DET) plots comparing the performance of the 9 methods in the absence of batch effects with 6 observations and 265 metabolites.B) Detection-error tradeoff (DET) plots comparing the performance of the 9 methods in the presence of batch effects with 12 observations and 265 metabolites.C) Detection-error tradeoff (DET) plots comparing the performance of the 9 methods in the absence of batch effects with 50 observations and 265 metabolites.D) Detection-error tradeoff (DET) plots comparing the performance of the 9 methods in the presence of batch effects with 100 observations and 265 metabolites.E) Detection-error tradeoff (DET) plots comparing the performance of the 9 methods in the absence of batch effects with 100 observations and 265 metabolites.F) Detection-error tradeoff (DET) plots comparing the performance of the 9 methods in the presence of batch effects with 200 observations and 265 metabolites.G) Detection-error tradeoff (DET) plots comparing the performance of the 9 methods in the absence of batch effects with 100 observations and 500 metabolites.H) Detection-error tradeoff (DET) plots comparing the performance of the 9 methods in the presence of batch effects with 200 observations and 500 metabolites.

By simulating negative control spike-in metabolites, it was also possible to explore the performance of additional methodologies which require such prior information. Remove Unwanted Variation (RUV)[11] and a control-based supervised variant on the Surrogate Variable Analysis (SVA)[27] were utilized as data normalization techniques in a two-stage procedure for analysis, coupled with LIMMA. Average Receiver-Operator Characteristic (ROC) curves and the associated areas under these curves (AUC) were calculated for the simulated sets and compared across methods for each operator combination, and across operator combination for each of the methods. Results are shown graphically in Fig 3. FAMT-DEF was unable to converge on a solution for 6/50 simulated data sets in the 100x500 no factor case. The remaining 44 simulated sets for which a solution was achieved were averaged over in the final comparison. analysis was performed on the false classification rates for each of the 9 methods. The miss rate for truly differentially abundant compounds, or the False Negative Rate (FNR), and the incorrectly discovered compounds, or False Positive Rate (FPR), were calculated for each of the methods given that the truly differential compounds were known in simulation. At varying FDR and posterior probability thresholds, the FNR and FPR were calculated, and the detection-error tradeoff (DET) curve for the methods are shown in Fig 4, scaled to a generally accepted FPR range of [0.0, 0.2]. In doing this, we provide a means for assessing the tradeoff in performance between two measures of negative error for various methods.

### Software

All computations were performed in R[32] version 3.3.1. (https://www.R-project.org). The heatmap in Fig 1E was generated by hierarchical clustering of the scaled metabolite abundance data using MetaboAnalyst 3.0[33].

## Results

### Operators induce major undesirable variation in metabolomics data

We used a metabolomics dataset in this study containing relative abundances of 265 metabolites after filtering, across a total of 24 samples obtained using LC-MS methods as described previously[5] (Methods). The samples were collected by four different operators—namely “A”, “X”, “D”, and “Z”. Each operator performed the metabolomics experiment on 6 samples in triplicates: three replicates in the untreated condition (control) and three replicates from samples treated with a drug (A and X treated samples with a different drug than D and Z). The biological goal in this experimental set-up was to discover metabolites that significantly change in abundance as a result of a drug treatment. Therefore, the operator factor is considered an unwanted effect whereas the drug treatment is the biological effect of interest in this experiment.

We first performed singular value decomposition (SVD) and factor analysis using RRmix to visualize the grouping of samples (Methods). Plot of the singular values reveal that the samples are clustered according to the operators (S1A Fig). Plots of the first four factor loadings extracted from the RRmix model analysis also confirm a distinct grouping of samples based on operators (S1B Fig), confirming the existence of a strong undesirable effect. For the subsequent analysis of differential metabolite abundances, we focused on the control and treated subset of the data from operators A and X with the exclusion of data from operators D and Z due to the difference in drug treatment.

Fig 1A and 1B illustrate the first two principal components (PCs) of the metabolomics datasets collected by operators A and X, respectively, where PC1 clearly separates control and treated samples in each case. The results show that when analyzed separately, samples collected by each operator cluster according to the treatment condition as expected. However, when all of the samples were combined and analyzed as a larger dataset, the main factor explaining variance was the operator effect, with PC1 separating operators A and X and PC2 separating samples with respect to the drug treatment (Fig 1C). The operator effect in this case is an example of undesirable variation in metabolomics data that can bias the subsequent differential abundance analyses. We observed that in the combined dataset, 56% of the overall variability is explained by PC1—a batch effect—and only 20% by PC2—the biological effect of interest (treatment with 2-deoxy-D-glucose (2DG)). Hierarchical clustering of the samples also confirmed that samples obtained from the same operator tend to group together more strongly than samples from the same biological condition (Fig 1D). In all cases, the three replicates from the same condition and the same operator were always clustered together as expected (Fig 1D).

### Simple statistical methods are able to detect biological effects in the absence of major batch effects

We next set out to perform differential abundance analyses using multiple approaches to find metabolites that are significantly affected by the drug treatment. Since 2DG directly targets a particular enzyme in the glycolysis pathway (Methods), metabolites in this pathway are expected to show substantial changes upon the drug treatment (Fig 2A). According to the underlying mechanism of action of 2DG in cells, we defined a set of 8 glycolytic metabolites that were expected to change in abundance upon the drug treatment as “positive controls”. This set of metabolites was then used to compare the performance of a number of differential abundance analyses methods listed in Table 1.

**Table 1:** Summary results of differential abundance analyses using various methods.

PERFORMANCE METRIC → METHOD ↓	No. of discoveries in absence of batch effects (total = 265)	No. of positive controls discovered in absence of batch effects (total = 8)	No. of discoveries in presence of batch effects (total = 265)	No. of positive controls discovered in presence of batch effects (total = 8)
**t-tests**	**49**	**5**	**24**	**2**
**LIMMA**	**115**	**6**	**19**	**4**
**PCA-LIMMA**	**158**	**5**	**119**	**7**
**SVA-LIMMA**	**152**	**7**	**114**	**7**
**FAMT**	**118**	**7**	**166**	**7**
**RRmix**	**39**	**6**	**42**	**7**

We first performed differential abundance analyses on samples from each operator separately using six statistical methods—the t-test, LIMMA, PCA coupled with LIMMA (PCA-LIMMA), SVA coupled with LIMMA (SVA-LIMMA), FAMT, and RRmix (Methods, S2A Fig). The t-test and LIMMA simply assess the significance of differences in mean abundance levels for each metabolite between the control and drug treated conditions without correction for potential batch effects. PCA and SVA normalize the data against unwanted variation before the analysis, and FAMT and RRmix use latent factor models to account for potential batch effects during the analysis (Methods). The total number of significant discoveries made by each method (at posterior probability of 0.9 for RRmix and 10% false discovery rate (FDR) for the other methods) is reported in Table 1 (see Methods for a discussion of the choice of posterior probability in RRmix). Results show that latent factor models as well as simpler methods such as the t-test make many common discoveries (S2B and S2C Fig), suggesting that they are able to perform equally well in the absence of any major batch effects (Table 1). Next, we compared the performance of the six methods using the positive control set of metabolites. Table 1 lists the number of positive controls that each of the methods was able to discover as significantly changing upon 2DG treatment (at posterior probability of 0.9 for RRmix and 10% FDR for the other methods). FAMT and SVA-LIMMA showed the best performances by detecting 7 of the 8 positive controls as significantly changed (Table 1). As expected, in the absence of major batch effects, even simple methods such as the t-test perform reasonably well in discovering metabolites with large changes in abundance (5 and 6 of the 8 positive controls were discovered by the t-test and LIMMA, respectively) (Table 1).

### Latent factor models are able to detect biological effects in the presence of batch effects

Next, we combined datasets obtained from operator A and operator X into one dataset and re-evaluated the performance of each method this time in the presence of an unwanted effect (S2D Fig). In the combined setting, the number of positive controls discovered by the t-test and LIMMA methods was lower than the other four methods (Fig 2B, Table 1). This confirms that in the presence of a batch effect, models that somehow account for unwanted hidden variability strongly out-perform models that ignore such variations. Consistent with this finding, the total number of discoveries made by the t-tests and LIMMA in this case was also much smaller than the other methods (only 11 metabolites were discovered as significantly changing by all 6 methods, whereas 21 additional metabolites—including 3 of the positive controls—were discovered by methods other than t-test and LIMMA; Fig 2C, Table 1). Results also suggest that the FAMT method may be discovering false positives that are not found significant by any of the other methods (Fig 2C, S2D Fig). Together, these results suggest that the RRmix algorithm is more suited to process metabolomics data with respect to both specificity and sensitivity (lower false positive and higher true positive rates).

### Latent factor models facilitate combining of datasets for increasing statistical power

In order to test the generalizability of our results and applicability of our method, we next attempted to perform a series of analyses using datasets of varying sizes. Due to the unfeasibility of obtaining large metabolomics datasets experimentally, we turned to simulated datasets. We used a series of simulations to further evaluate the performance of 9 different methods. These methods include the individual t-tests, LIMMA, PCA coupled with LIMMA (PCA-LIMMA), unsupervised SVA coupled with LIMMA (UNSUPSVA-LIMMA), negative-control supervised SVA coupled with LIMMA (SUPSVA-LIMMA), RUV coupled with LIMMA (RUV-LIMMA), FAMT with (FAMT-NBF) and without (FAMT-DEF) specifying the number of latent factors to be estimated, and RRmix). Receiver operating characteristic (ROC) and detection error trade-off (DET) curves for multiple simulation settings were generated using the results. In general, it is expected that increasing sample size would increase the statistical power associated with identifying true positives. However, to mimic the real dataset, we introduced a batch effect in the larger sample size simulations whereas no batch effects were present in the smaller sample size cases (Methods).

The area under the curve (AUC) associated with the t-test decreases by 30% and 15% when sample size increases from 6 to 12 or from 50 to 100, respectively (Fig 3A–3D), indicating that the unwanted batch effect is confounding the true biological effect causing t-tests to perform worse despite the increase in sample size. In contrast, latent factor models are able to adjust for the unwanted variation and perform better with larger sample sizes in the presence of batch effects (for instance, RRmix AUC increases by 4% and 0.4% when sample size increases from 6 to 12 or from 50 to 100, respectively; Fig 3A–3D). This finding shows that if the appropriate algorithms are used to analyze metabolomics data, researchers can benefit from combining multiple data sets by gaining higher statistical power. We confirm using simulations that the t-tests and LIMMA perform as well as the other seven methods in the absence of batch effects (Fig 3A, 3C, 3E and 3G), while in the presence of batch effects, RRmix, FAMT, and UNSUPSVA -LIMMA significantly outperform methods that fail to account for unwanted variations (t-test and LIMMA) as well as alternative normalization methods (PCA-LIMMA, SUPSVA-LIMMA, and RUV-LIMMA) (Fig 3B, 3D, 3F and 3H).

As shown in the larger dataset simulations, accounting for latent variation facilitates gains in performance of all of the methods tested (Fig 3E–3H). However, in the presence of batch effects, most methods do progressively worse with decreasing sample sizes, while RRmix, FAMT, and UNSUPSVA-LIMMA maintain highest sensitivity levels (Fig 3B, 3D, 3F and 3H). These methods perform very well comparatively, and have the added benefit of not requiring prior knowledge of batch effect sources or need for negative controls.

### RRmix outperforms FAMT with respect to specificity

The RRmix approach is shown to benefit from imposing sparsity on the model directly. This is done through the mixture component, with a prior assumption placed on the *β*_*g*_’s with the *b*_*g*_’s. By modeling the large number of null effects directly, RRmix differs from FAMT, which relies on heavy FDR thresholding after model selection. In simulation, RRmix was robust to changes in posterior probability cutoff, while the number of metabolites FAMT found to be significantly different between ‘treatment’ and ‘control’ groups depended greatly on FDR threshold selection. S3 Fig shows the ranked null probabilities for each of the 265 metabolites from the LC-MS dataset as calculated by both RRmix and FAMT. There is a steep drop-off between calculated null and non-null metabolites for RRmix, while the null probabilities for FAMT gradually decrease with each successive rank. Overall, this shows that methods such as RRmix are better suited to correctly classify metabolites of interest as significant or not, even with changes in significance threshold cutoff, due to their consideration of sparsity of the effects directly in the model (Methods). Notably, an analysis of the detection error tradeoff reveals that RRmix yields the greatest performance with respect to false classifications of any of the methods in all simulations (Fig 4A–4H). We emphasize here that RRmix is able to outperform other methods despite the lack of incorporation of any prior knowledge or additional controls in this algorithm, illustrating the appeal of RRmix over previously proposed alternatives.

## Conclusions

Similar to other types of high-dimensional data, the distinction between hidden undesirable variation and true biological variation in metabolomics data is crucial in order to ensure reproducibility and draw conclusions from experimental results. Some of the approaches frequently used to address this issue either impose additional experimental burden such as the use of multiple internal standards, or rely on prior knowledge of undesired effects such as the distribution of signal intensities, feature-specific drift patterns, or the determination of a set of metabolites as controls that are robust to batch effects.

Here, we illustrate that latent factor models can reliably remove undesirable variability from metabolomics data while preserving true biological effects. The main advantage to utilizing such algorithms is that they rely solely on mathematical methods to correct for batch effects at the data analysis step without prior knowledge about the nature of the batch effects. Therefore, these algorithms are robust, and can be applied to pre-existing metabolomics data as well as future studies. In the family of latent factor models, we introduced the RRmix algorithm and show that it efficiently handles batch effects in metabolomics data as well as simulated data, and it outperforms alternative approaches. We believe that the field will greatly benefit from application of these findings and implementation of the latent factor models introduced here. Wider implications of this study include the applicability of the RRmix model to other fields of study that handle high-dimensional biological data. Genomics, transcriptomics, proteomics, and other such disciplines often focus on detecting significant differences in mean measurable units from arrays of size *n* × *p* with *n* << *p*. We should note that although RRmix uses the entirety of a given dataset in one step for the model fitting and estimation procedure, it is different from traditional multivariate methods that consider interactions between different variables (in this case metabolites) into their models. As discussed in the manuscript, the main function of RRmix is detecting differential expression in individual variables, however, it is plausible to try to expand this framework to multivariate analyses in the future to involve testing biologically meaningful groups of metabolites. We believe that RRmix model will also perform well in analyzing high dimensional datasets other than metabolomics data, although this remains to be validated.

## Supporting information

S1 Text Supplementary information on simulation details. (DOCX)

S1 Fig Dataset visualization. A) Plots denotes the pairwise sequential comparison of the factor loadings from singular value decomposition (SVD), with the left showing the plots produced with the first factor loading on the x-axis and the second factor loading on the y-axis, the middle denoting the plots of the second factor loading on the x-axis and the third factor loading on the y-axis, and the right plotting the third factor loading on the x-axis and the third factor loading on the y-axis.B) Plots are organized similarly to part (A) with the factor loadings from the RRmix model.
(TIFF)

S2 Fig Method comparisons. A) Diagram depicting the approach used to compare the performance of the four methods with respect to detecting metabolite abundance changes upon drug treatment using data collected by individual operators (no major batch effects present).B) Total number of significant discoveries made by each method using metabolomics data from operator “A” (RRmix p = 0.9; FDR 10%).C) Total number of significant discoveries made by each method using metabolomics data from operator “X” (RRmix p = 0.9; FDR 10%).D) Diagram depicting the approach used to compare the performance of the four methods with respect to detecting metabolite abundance changes upon drug treatment using metabolomics data in the presence of a batch effect—operator.E) Venn diagram comparing total number of discoveries made by each of the methods in the combined dataset (RRmix p = 0.9; FDR 10%).
(TIFF)

S3 Fig FAMT vs. RRmix. A) Plot showing the distribution of null probabilities for the 265 metabolites from the LC-MS metabolomics dataset (ranked in reverse-significance order) as calculated by RRmix and FAMT.
(TIFF)
