# Supplementary material for: RRmix: A method for simultaneous batch effect correction and analysis of metabolomics data in the absence of internal standards
Source: PLoS One. 2017 Jun 29;12(6):e0179530. doi: 10.1371/journal.pone.0179530 (PMC5491020; doi:10.1371/journal.pone.0179530)
Supplement: S1 Text — (DOCX) [file pone.0179530.s001.docx]

S1 Text

**Simulations**

Within each of the 50 simulated data sets in each set, the compound-specific error variance was simulated from an $Inverse-Gamma(A, B)$ distribution with shape and scale parameters set equal to $(3,1)$ for each of $G=265$ simulated compounds, reflecting the number of metabolites tested in the original data set^28^. The compound-specific latent indicator was then simulated as a random Bernoulli with parameter $p=0.05$ such that 5% of the metabolites would have non-null status between treatment and control groups. The $\beta_{g}$ from the RRmix model specification were simulated given the model assumptions with $\psi=0.5$. The variance components for the random bivariate-normal were set to $\sigma_{0}^{2}=0.055$ and $\sigma_{1}^{2}=0.23$ based on estimates from the original data set. Multiplying this vector by the design matrix $X$ gives the additive component of the simulated responses for the known portion of the data. The latent loadings for the synthetic batch effects were given by randomly permuting a set of four linear contrasts with random noise added to each matrix element simulated from a $N(0,0.01)$ distribution. The factor coefficients were simulated from a $N(0,1)$ distribution and multiplied with the loading matrix to give the additive component of the simulated response for the hidden portion of the data. The estimated grand mean of 13 from the data was added to the simulated responses. Finally, random noise was added to each response following a $N(0,\sigma_{g}^{2})$distribution, with the $\sigma_{g}^{2}$ simulated previously. In addition, 5% of the metabolites are simulated so that they resemble negative control metabolites as used previously^10^. Data simulated in the single operator case followed similarly, with no latent factor component and sample size n=6. Due to low sample sizes in the study data, as well as the relatively low metabolite counts, three additional sets of simulations were performed with simulated datasets consisting of 50 and 100 observations on 265 metabolites, 100 and 200 observations on 265 metabolites, and 100 and 200 observations on 500 metabolites, with each pair of observations pertaining to no unwanted variation and two latent factors respectively. As before, the simulation parameters were estimated using the RRmix model analysis on the original data set, and 5% of the metabolites are truly differentially abundant between the samples designated treatment and control.
