# Supplementary figures and images for: RRmix: A method for simultaneous batch effect correction and analysis of metabolomics data in the absence of internal standards
Source: PLoS One. 2017 Jun 29;12(6):e0179530. doi: 10.1371/journal.pone.0179530 (PMC5491020; doi:10.1371/journal.pone.0179530)

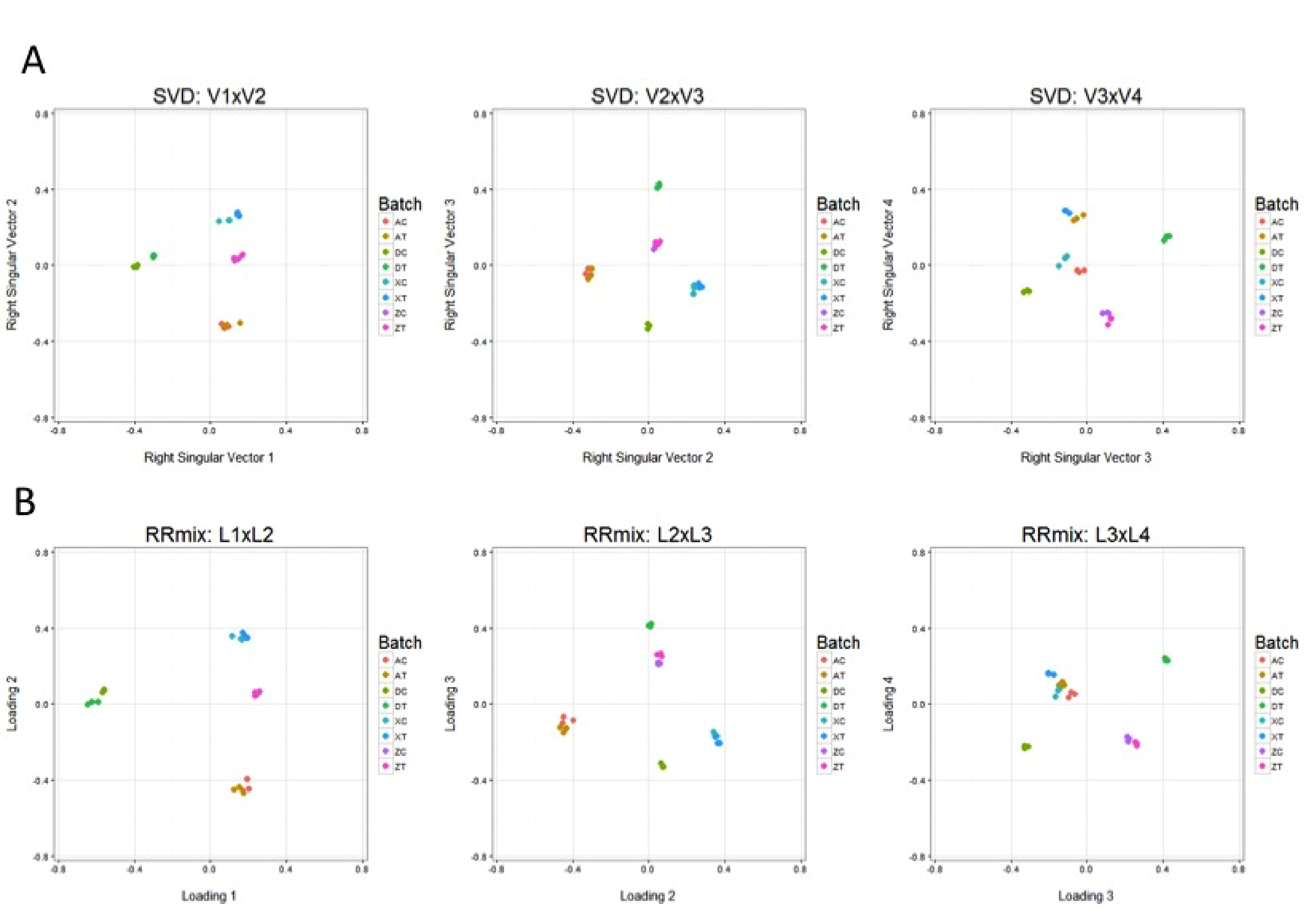

Supplement: S1 Fig — A) Plots denotes the pairwise sequential comparison of the factor loadings from singular value decomposition (SVD), with the left showing the plots produced with the first factor loading on the x-axis and the second factor loading on the y-axis, the middle denoting the plots of the second factor loading on the x-axis and the third factor loading on the y-axis, and the right plotting the third factor loading on the x-axis and the third factor loading on the y-axis.B) Plots are organized similarly to part (A) with the factor loadings from the RRmix model. (TIFF) [file pone.0179530.s002.tiff]

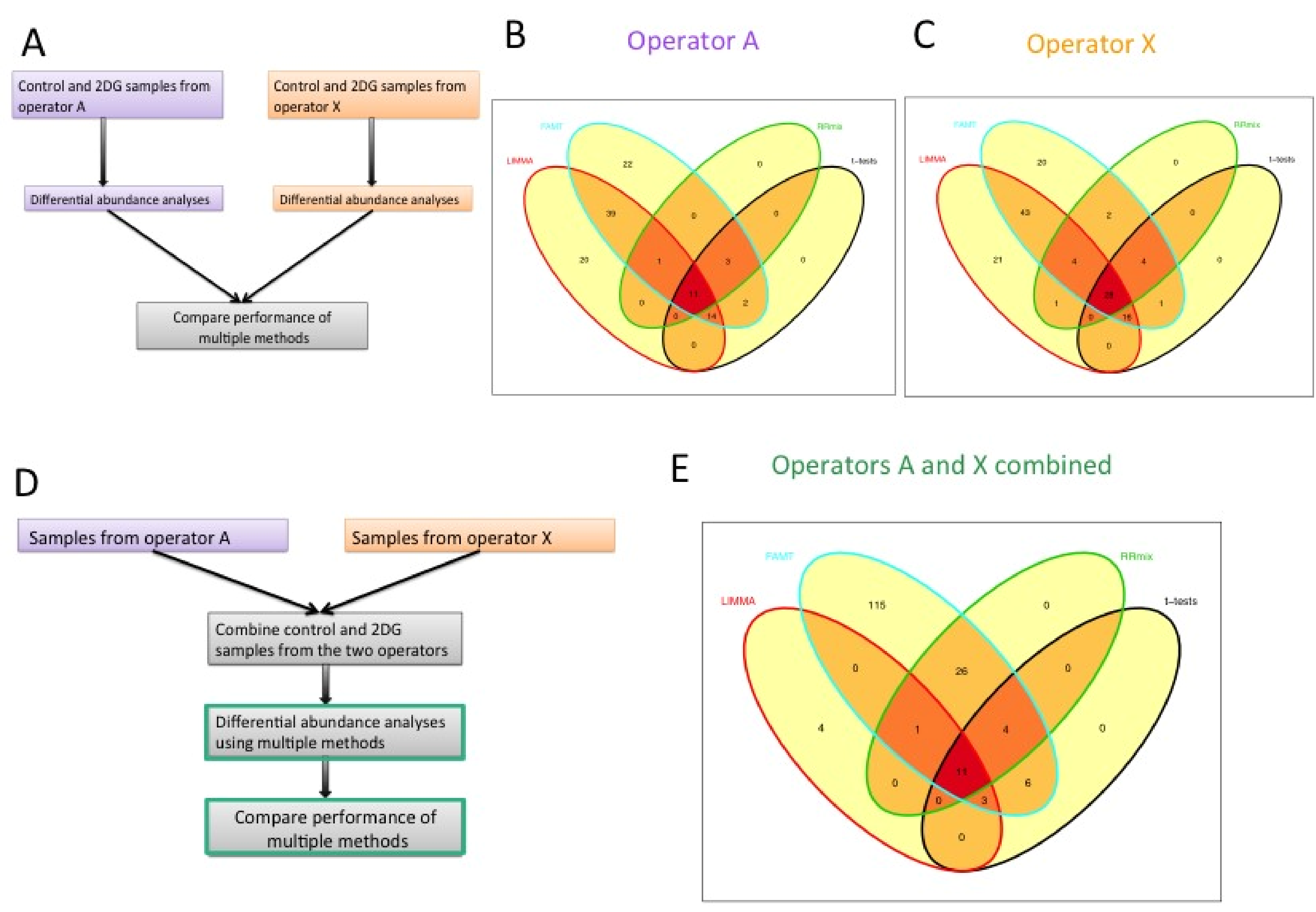

Supplement: S2 Fig — A) Diagram depicting the approach used to compare the performance of the four methods with respect to detecting metabolite abundance changes upon drug treatment using data collected by individual operators (no major batch effects present).B) Total number of significant discoveries made by each method using metabolomics data from operator “A” (RRmix p = 0.9; FDR 10%).C) Total number of significant discoveries made by each method using metabolomics data from operator “X” (RRmix p = 0.9; FDR 10%).D) Diagram depicting the approach used to compare the performance of the four methods with respect to detecting metabolite abundance changes upon drug treatment using metabolomics data in the presence of a batch effect—operator.E) Venn diagram comparing total number of discoveries made by each of the methods in the combined dataset (RRmix p = 0.9; FDR 10%). (TIFF) [file pone.0179530.s003.tiff]

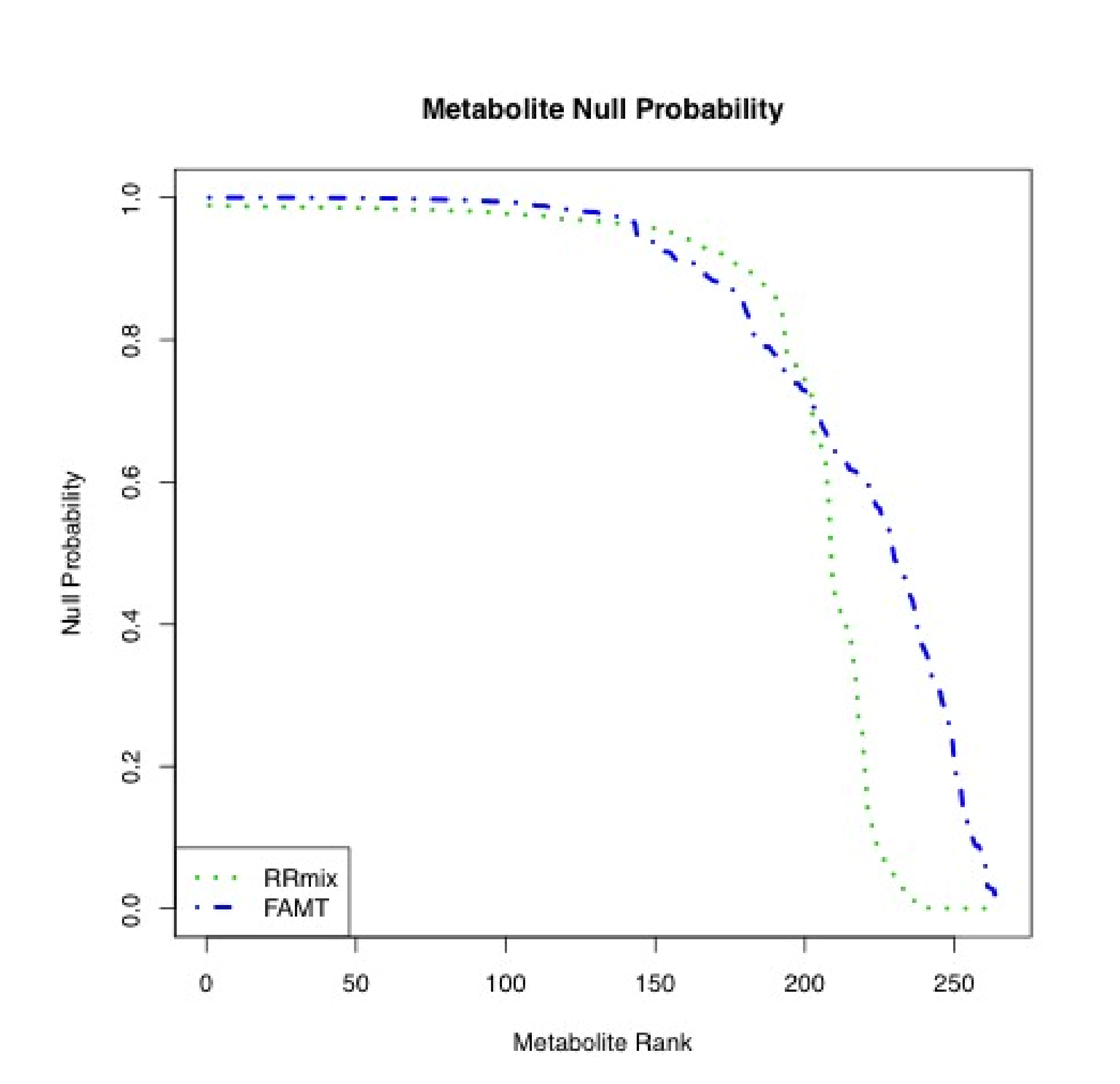

Supplement: S3 Fig — A) Plot showing the distribution of null probabilities for the 265 metabolites from the LC-MS metabolomics dataset (ranked in reverse-significance order) as calculated by RRmix and FAMT. (TIFF) [file pone.0179530.s004.tiff]
